# Editorial: Sport and exercise: challenges and perspectives in mental health

**DOI:** 10.3389/fpsyt.2023.1266672

**Published:** 2023-08-24

**Authors:** Thomas Wenzel, David Baron

**Affiliations:** ^1^Department of Psychiatry, Scientific Section for Exercise and Sport Psychiatry, World Psychiatric Association (WPA), Geneva, Switzerland; ^2^Center for Mental Health and Sports, Western University of Health Sciences, Pomona, CA, United States

**Keywords:** sport, exercise, public health, athlete, stress, ethics

Sport is a key area of public interest and has again become an important issue in public health and in the life of many individuals. Already in ancient cultures, such as that of Greece, it played an important role in public life and the reputation of community members and communities. A healthy body and healthy mind were mostly seen as closely related. In recent years, the link between physical and mental health has consequently also become a focus in medicine and in public mental health. The term “sports” is used to cover an extensive range of activities, even including competitive card and internet-based games, though this has not gone undisputed. The newly emerging field of Sport Psychiatry covers a wide range of areas of concern, from the health and performance of competitive athletes to amateur sports and the psychology of spectators and other indirect participants. It also has to cover considerations such as transcultural factors as sport participation is globalized and taken up by all ethnic groups, as explored by Yim et al. in this collection of articles. The understanding that sport can be emotionally rewarding has been extended to the significant health and mental health risks present in some forms of mainly, but not exclusively, high-performance and competitive sports, such as eating disorders, brain trauma (1), posttraumatic spectrum disorders, doping, depression, and suicide (2). The suicide of the German national goalkeeper Enke has drawn attention to these special and related, but until then largely underrated, risks and has led to the creation of special sport psychiatry services in German university hospitals. This also reflects the frequently underestimated complexity of treatment and interventions in sport psychiatry as it is necessary to consider not only the culture but also the specific sport-related subculture of athletes and the special needs of athletes in regard to performance, as explored (3) and by Kvillemo et al. Young adults in sports need special attention, as confirmed for the special situation of college athletes by Moore et al. and Du et al.^1^

A special, encouraging related field is that of the use of sport and exercise as tools to promote general and psychological health and even assist in the treatment of patients with mental health problems, as Yokoyama et al. has demonstrated in regard to the special Japanese challenge of Hikikomori adults and (4) and Du et al. for Chinese college students, who are under great exam stress in a highly demanding school system. Lifestyle psychiatry as a new specialty in this context underlines the advantages of embedding sport and exercise into everyday life to promote not only physical but also mental health—in this Research Topic explored by Almeida et al. and Yuan et al.—and even in forensic populations, as demonstrated by Reimer and Kanning. The European Union has, for example, recently included a major funding focus on sport and exercise to reach a larger audience of the general public and offer adequate stimulus to sport in everyday life. The EU argues on different levels to justify this approach on their website, stating that “sport represents an integral part of the lives of millions of Europeans. Support for sport builds community cohesion, grows social inclusion and leads to an enhanced sense of European identity. Sport is also a key facet of Member States' and the larger European economies; the sector employs millions of European citizens, and adds billions in revenue”^2^ —the last point in regard to the economy being an important but not always positive aspect in this context, not only in Europe and the US.

A new field of concern is that of the protection of human rights, medical ethics, and ethical and healthy sport practice. This includes examining the widespread use of doping with or without the expressed knowledge and consent of athletes (5, 6) and the interference of high-performance sports—fueled by the ambition not only of athletes but also parents, trainers, and coaches or by political considerations—with the development of children and young adults and, later, with a healthy lifestyle. Racism (7, 8), sexual abuse (9–11), persecution, and abuse of athletes in the political context are part of a larger range of problems. These factors must be also addressed and considered as key elements in healthy sports.

Challenges such as racism and violence, associated in many situations with groups of spectators but also with some athletes, or the risks ranging from habitual passive consumption of sports events to addiction patterns still need to be covered in future research.

It should be finally considered that healthy exercise and sports require the coordinated efforts of all relevant professions, families, friends and caregivers, team doctors, and finally, of course, the athletes themselves, who have to learn and respect that any sports activity must respect health and human rights. Legislators and sport officials and their organizations have to implement strong and effective measures to keep sport ethical and healthy; this has been frequently neglected in an era where political and other abuses of athletes, and the predominance of financial and publicity interests, have become closely associated with many sports fields.

## Author contributions

TW: Writing—original draft. DB: Writing—review and editing.

## Footnotes

^1^All citations not otherwise marked, are part of this article collection.

^2^https://sport.ec.europa.eu/sport-in-the-eu (accessed June 20, 2023).
